# Risk Factors Leading to Enucleation or Evisceration in Infectious Endophthalmitis

**DOI:** 10.3390/jcm11113145

**Published:** 2022-06-01

**Authors:** Ambar N. Lugo Merly, Lorena A. Montalvo Toledo, Guillermo A. Requejo, Alexander Meléndez, Samuel Álvarez, Andrés López, Radames Ríos, Victor M. Villegas, Armando L. Oliver

**Affiliations:** Department of Ophthalmology, University of Puerto Rico School of Medicine, Medical Sciences Campus, San Juan, PR 00936, USA; ambar.lugo@upr.edu (A.N.L.M.); lorena.montalvo2@upr.edu (L.A.M.T.); grequejo70@gmail.com (G.A.R.); alexander.melendez16@hotmail.com (A.M.); samu.alvarez@gmail.com (S.Á.); andres.lopez15@upr.edu (A.L.); radames.rios@upr.edu (R.R.); victor.villegas@upr.edu (V.M.V.)

**Keywords:** endophthalmitis, panophthalmitis, evisceration, enucleation

## Abstract

Endophthalmitis treatment consists of intravitreal antibiotics injections and, in selected circumstances, pars plana vitrectomy. However, severe or refractory cases may require an enucleation or evisceration (ENEV). Our study seeks to identify risk factors leading to enucleation or evisceration in patients with infectious endophthalmitis. A retrospective chart review of subjects with a clinical diagnosis of infectious endophthalmitis was undertaken. The affected eyes were stratified into groups: those that underwent ENEV and those in which the eyeball was preserved (EP). The groups were compared using statistical analyses. In total, 69 eyes diagnosed with infectious endophthalmitis were included in the study. There was a higher frequency of exogenous infectious endophthalmitis in the ENEV group versus the EP group. Postsurgical infectious endophthalmitis was lower in the ENEV than in the EP group. A visual acuity of no light perception was more common in the ENEV compared to the EP group. Panophthalmitis was more frequent in the ENEV versus the EP group. Our findings suggest that eyes with endophthalmitis presenting with a visual acuity of no light perception, panophthalmitis, or exogenous etiology have a higher risk of requiring ENEV. In addition, eyes with a postsurgical etiology may be at a lower risk of requiring ENEV.

## 1. Introduction

Endophthalmitis is an inflammatory process affecting the eye’s inner structures; it is frequently associated with infection [1,2,3]. The most common risk factors include trauma, intraocular surgery, and hematogenous dissemination of infectious microorganisms to the eye [4,5,6,7,8].

Despite advances in vitreoretinal surgery, the prognosis of endophthalmitis tends to be poor [9]. The literature regarding risk factors associated with enucleation or evisceration (ENEV) in infectious endophthalmitis continues to be limited. Some studies have shown that corneal ulcer-related infectious endophthalmitis, endogenous or traumatic endophthalmitis, pseudophakia, age (i.e., being of a relatively advanced age), poor initial visual acuity, an immunocompromised state, and delayed intervention are associated with a higher probability of requiring ENEV [2,9,10,11].

The purpose of our study was to identify the risk factors that led to ENEV in subjects who were diagnosed with infectious endophthalmitis at the University District Hospital (UDH), the only tertiary referral hospital in Puerto Rico.

## 2. Materials and Methods

A retrospective chart review from August 2015 through August 2020 was undertaken using records pulled from the databases of the Medical Services Administration of Puerto Rico (ASEM, by its initials in Spanish), the UDH, and the outpatient clinics of the University of Puerto Rico Department of Ophthalmology. All the charts with a diagnosis of infectious endophthalmitis or panophthalmitis were analyzed.

Subjects with a clinical diagnosis of infectious endophthalmitis (or panophthalmitis) were included. All the subjects had undergone a complete ophthalmologic exam. Demographic and clinical characteristics including age, sex, date of presentation, past medical history, visual acuity at presentation, physical findings, and primary and secondary treatments were recorded.

The different endophthalmitis etiologies in our study were categorized as exogenous, postsurgical, post-traumatic, or endogenous, according to the history and clinical findings obtained from the chart review. Those eyes whose endophthalmitis was secondary to an infectious corneal keratitis or complicated bullous keratopathy were categorized as exogenous etiology. A postsurgical etiology was assigned if the endophthalmitis occurred as a complication of a glaucoma or cataract surgery, pars plana vitrectomy, corneal transplant, or exposed infected suture. The post-traumatic endophthalmitis etiology included patients who presented with endophthalmitis secondary to a traumatic open globe or presented with an intraocular foreign body. Patients whose endophthalmitis occurred concomitant to a confirmed or suspected systemic infection source were categorized as having endogenous etiology. If no etiology was elucidated, these were classified as undetermined.

Microbiology and pathologic specimens were reviewed and analyzed. The exclusion criteria were not meeting the clinical diagnosis of infectious endophthalmitis and having an incomplete medical record. Predisposing immunocompromised states including and/or resulting from diabetes mellitus, HIV, immunosuppressive medications, cancer chemotherapy, and high-dose systemic corticosteroids were also reviewed.

The subjects that met the diagnostic criteria for infectious endophthalmitis or panophthalmitis were divided into two groups: subjects whose affected eye underwent ENEV and those whose eye was preserved (EP). The frequencies of the selected data within both groups were compared and statistically analyzed. The chi-square test and Fisher’s exact test were used to assess differences between categorical variables. Welch’s *t*-test was used to analyze the mean differences of continuous variables. All statistical analyses were performed using R Studio open-source software and Microsoft Excel. *p* values less than 0.05 were considered statistically significant. The corresponding odds ratios (ORs) and 95% confidence intervals (CIs) were calculated to identify potential risk factors for ENEV.

## 3. Results

A total of 69 eyes of 69 subjects with a clinical diagnosis of infectious endophthalmitis or panophthalmitis were included in our study. The median age was 70 years (range 10–95 years). Twenty-eight (40.58%) were women, and 41 (59.42%) were men. Fourteen (20.29%) eyes had a diagnosis of panophthalmitis, and 55 (79.71%) had a diagnosis of endophthalmitis. Postoperative endophthalmitis was present in 26 eyes (37.68%). Exogenous endophthalmitis was present in 23 eyes (33.33%). Post-traumatic endophthalmitis was present in 10 eyes (14.49%). Endogenous endophthalmitis was present in nine eyes (13.04%). One eye (1.45%) had an undetermined etiology. Table 1 details the study population’s characteristics.

The type of surgeries leading to endophthalmitis in the postsurgical subgroup included 11 cataract surgeries (42.31%), 9 trabeculectomies (34.62%), 3 intravitreal injections (11.54%), 1 suture removal (3.85%), 1 pars plana vitrectomy (3.85%), and 1 corneal transplant (3.85%).

The subjects’ comorbidities included diabetes mellitus (62.32%), being in an immunosuppressive state (4.35%), having had a recent systemic surgery (2.90%), and having a history of intravenous drug use (2.90%).

Visual acuity at initial presentation included no light perception (NLP) in 17 eyes (24.64%), light perception (LP) in 19 eyes (27.54%), hand motion in 15 eyes (21.74%), and counting fingers or better in 15 eyes (21.74%); in 3 eyes (4.35%), visual acuity was not determined.

Positive microbiology isolates were present in 31 eyes (44.93%). Thirty isolates (96.77%) were bacterial and 1 was fungal (3.23%). Twenty-two isolates (73.33%) were Gram-positive, 7 (23.33%) were Gram-negative, and 2 (6.67%) were Gram-indeterminate. One eye had two positive isolates.

Forty-six eyes (66.67%) received a vitreous tap with an injection of intravitreal antibiotics as the initial treatment, 2 (2.90%) underwent a pars plana vitrectomy as the initial treatment, 2 (2.90%) received subconjunctival antibiotics prior to enucleation, and 18 (26.09%) underwent ENEV as the initial treatment; in 1 (1.45%) eye, the initial treatment was not recorded. Of the 46 patients that received intravitreal antibiotics as their initial treatment, 23 (50.00%) underwent a pars plana vitrectomy as an additional treatment due to a lack of clinical improvement after intravitreal therapy.

Thirty-two (46.38%) patients (mean age, 72 years) underwent ENEV of a single eye. Thirty-seven (53.62%) eyes were preserved, their possessors having a mean age of 66 years. Differences in mean age frequencies between the ENEV and EP groups were not statistically significant (*p* = 0.071). In the ENEV group, half of the subjects were females (16, 50.00%). Most of the subjects in the group whose eyes were conserved were males (25, 67.57%); 12 were females (32.43%). Differences in sex frequency between the ENEV and EP groups were not statistically significant (OR: 0.485; *p* = 0.216). Table 2 details these findings.

Exogenous endophthalmitis was significantly more frequent in the ENEV group (56.25%) than in the EP group (13.51%) (OR: 7.946; *p* = 0.0004664). In contrast, postsurgical endophthalmitis was less frequent in the ENEV (21.88%) group than in the EP group (51.35%) (OR 0.271; *p* = 0.02317). There was no significant difference in the post-traumatic and endogenous etiology between the groups (OR: 1.182, and *p* = 1; OR: 0.291, and *p* = 0.161, respectively). History of diabetes, immunosuppression, and IVDA history were not significantly different between the two groups (OR: 0.794, and *p* = 0.8257; OR: 0.553, and *p* = 1; and OR: 1.098, and *p* = 1, respectively).

The ENEV group had 24 (75.00%) subjects whose time of symptom onset to presentation lasted more than 2 days, while 6 (18.75%) subjects had a time to presentation of less than or equal to 2 days. In the EP group, 20 (54.05%) subjects had a time to presentation of more than two days, and 16 (43.24%) subjects had a time to presentation of equal to or less than 2 days. However, these differences were not statistically significant (OR: 0.318; *p* = 0.066). In three subjects, the time of symptom onset to presentation was undetermined, so their data for this element were not included in the statistical analysis.

The ENEV group had a significantly larger proportion of eyes with an initial visual acuity of NLP than did the EP group, with 15 (46.88%) and 2 (5.41%), respectively (OR: 17.832; *p* = 0.00003). Furthermore, the ENEV group had a smaller proportion of eyes with light (21.88%) and hand-motion perception (12.59%) compared to the EP group (32.43% and 29.73%, respectively); however, these differences were not statistically significant (OR: 0.667, and *p* = 0.641; OR: 0.38, and *p* = 0.1497, respectively). Similarly, the proportion of eyes with a visual acuity of counting fingers or better was smaller in the ENEV group compared to the EP group (9.38% vs. 32.43%, respectively) (OR: 0.2453; 95% CI: 0.0397–1.06; *p* = 0.04396). The proportion of subjects with a diagnosis of panophthalmitis was significantly higher in the ENEV group (37.50%) than in the EP group (5.41%) (OR: 10.15; *p* = 0.001836).

The number of positive cultures was similar between the two groups, with 16 (50.00%) positive cultures in the ENEV group and 15 (40.54%) in the EP group (OR: 1.458; *p* = 0.5857). All the positive cultures in the ENEV group were bacterial (16, 50.00%), and the EP group had 14 (37.84%) bacterial isolates (OR: 1.631; *p* = 0.4396). The EP group had one (2.70%) culture positive for a fungal pathogen. The ENEV group had 10 (31.25%) Gram-positive isolates, and 12 (32.43%) isolates in the EP group were Gram-positive. The ENEV group had a total of five (15.63%) Gram-negative isolates, and the EP group had a total of two (5.41%) Gram-negative isolates. One eye in the EP group had more than one isolate in the same culture, of which one was Gram-positive and one Gram-negative. Differences in Gram staining were not statistically significant between the two groups (OR: 0.346; *p* = 0.3898). Both groups had one isolate each that was Gram-indeterminate.

Of the 32 patients in the ENEV group, 14 (43.75%) received additional treatments prior to the ENEV. These prior treatments consisted of 11 (34.38%) intravitreal injections, 1 (3.13%) pars plana vitrectomy, and 2 (6.25%) administrations of subconjunctival antibiotics. The remaining 18 (56.25%) patients in the ENEV group underwent ENEV as their initial treatment.

## 4. Discussion

Our institutional treatment protocol for endophthalmitis consists of an immediate vitreous tap and the intravitreal injection of vancomycin and ceftazidime. For eyes with a history of trauma, a suspected endogenous etiology, or clinical findings suggestive of fungal etiology, we add antifungal medication such as voriconazole or amphotericin B. Open globes (eyes) with retinal or choroidal detachment are typically not injected intravitreally. Eyes presenting with LP vision or in which a fungal etiology is suspected, or those that are refractory to therapy, are considered for a pars plana vitrectomy. However, whether a pars plana vitrectomy is performed depends on factors such as corneal clarity, other media opacities, and the absence of systemic contraindications for surgery. Some eyes presenting with NLP visual acuity, for which the prognosis for eye preservation is deemed poor, do not receive intravitreal antibiotics, and instead are initially treated with ENEV. Our study suggests that eyes with infectious endophthalmitis presenting with a visual acuity of NLP, panophthalmitis, or exogenous etiology have a higher risk of requiring ENEV.

One major finding in our study was the association of exogenous endophthalmitis with ENEV. Lu et al. found that corneal ulcer–related infectious endophthalmitis was significantly more frequent in the ENEV group (50%) than in the salvage group (4.4%) [9]. In their study, Dave et al. found that the most common etiology of endophthalmitis necessitating evisceration was corneal ulcer-related (58%), accounting for half of all cases [2]. The progression of an infectious corneal ulcer to endophthalmitis could be attributed to delayed diagnosis and treatment, a delay in culture results, the use of topical steroids, a pre-existing ocular pathology, a previous ocular surgery, dementia, nursing home residential care, or systemic immunosuppression [9,12]. Corneal opacity and poor evaluation of the posterior pole may obstruct the view for a pars plana vitrectomy combined with temporary keratoplasty [9]. A patient with low socioeconomic status, poor accessibility to subspecialty care, lack of understanding of the severity of the condition, and/or poor adherence to treatment may also delay said treatment, decreasing the possibility of salvaging the affected eye [13]. The organisms associated with corneal ulcers may also lead to worse outcomes once intraocular infection develops.

Due to the high virulence of certain microbes, severe corneal ulcer–related endophthalmitis has been associated with poor visual outcomes. Streptococcal species, Staphylococcus aureus, Pseudomonas aeruginosa, and fungal isolates are known common pathogens associated with corneal ulcer-related infectious endophthalmitis [12,14,15]. Various studies have found that the organisms most likely to lead to infectious endophthalmitis are Gram-positive microorganisms [2,16].

In a cohort study in Melbourne, Australia, O’Neill et al. found that 83.9% of corneal ulcer-related infectious endophthalmitis cultures were Gram-positive and 14% were Gram-negative [12]. Streptococcal species was found to be the most frequent (32.4%), followed by Pseudomonas aeruginosa (29.7%) and Staphylococcus aureus (21.6%) [12]. Additionally, this study found that the most common pathogen in patients requiring ENEV was Pseudomonas aeruginosa (39.1%), followed by streptococcal species (30.4%). Eyes with both pathogens had an ENEV rate higher than 80% [12]. Otherwise, Dave et al. reviewed 791 cases of endophthalmitis that underwent evisceration; they reported that Streptococcus pneumoniae (17.52%) was the most common Gram-positive bacterial isolate, followed by Aspergillus (14.95%) and Pseudomonas (12.11%) [2]. Despite the differences in the studies, both streptococcal species and Pseudomonas aeruginosa were proven to be highly virulent microbes known to cause severe infectious corneal ulcers, which commonly have poor visual acuity outcomes [12]. However, we found a higher incidence of Gram-negative microbes within our ENEV group. The poor prognosis of these cases is not limited to the type of bacteria; instead, it is attributed to the vigorous inflammatory response that the bacteria cause in the eye, which results in damage to the eye and worsens the visual acuity of the patient [17]. Furthermore, the high virulence and antibiotic resistance of the pathogens associated with infectious corneal ulcer-related endophthalmitis compared to postsurgical and post-traumatic endophthalmitis leads to a poor prognosis in the exogenous endophthalmitis [15].

Postoperative infectious endophthalmitis has been associated with better visual outcomes compared to those of other etiologies [5]. Lu et al. found that postoperative endophthalmitis was a significantly less frequent etiology in ENEV [9]. Similarly, we found that eyes with postoperative endophthalmitis had a decreased risk of requiring ENEV. Yannuzzi et al. found that 24 of 63 (38%) eyes diagnosed with acute onset endophthalmitis after clear corneal cataract surgery achieved a visual acuity of at least 20/40 after treatment [18]. The improved visual prognosis in postoperative endophthalmitis can be attributed to the various methods of endophthalmitis prophylaxis applied during and after ophthalmic surgery as well as to the pathogens associated with the infection. The most common pathogen associated with postsurgical endophthalmitis is the staphylococcus species [15,19]. In their study, Ramakrishnan et al. found that isolated organisms from postsurgical endophthalmitis have a higher antibiotic susceptibility rate than do other etiologies [15]. The pathogens commonly associated with postoperative endophthalmitis (Gram-positive, coagulase-negative staphylococci) seem to cause a less severe infection compared with Gram-negative and other pathogens, the latter being less common in this subgroup [20]. Less virulent pathogens and intraoperative prophylaxis could both contribute to a decreased rate of ENEV in postoperative endophthalmitis cases [15,21]. Additionally, it is important to note the significance of the timing of the physician evaluation and diagnosis of endophthalmitis. In postoperative endophthalmitis, the most common presentation is within seven days of surgery [20]. This acute presentation means that in postoperative endophthalmitis, earlier physician evaluation is more typical. Gao et al. found that patients with postoperative endophthalmitis and post-traumatic endophthalmitis had a shorter time to visit the physician when compared with corneal ulcer-related and endogenous endophthalmitis [5]. In the aforementioned study, both etiologies were associated with improved visual outcomes when compared to corneal ulcer-related and endogenous etiologies [5]. Postoperative follow-up physician visits are routinely done, leading to earlier diagnosis and treatment of postoperative endophthalmitis, increasing the likelihood of a better outcome.

A severe complication of endophthalmitis, panophthalmitis involves an infectious process that extends to the orbital contents [22]. Ang et al. found that panophthalmitis was a risk factor for poor visual outcomes in patients with endogenous panophthalmitis caused by Klebsiella pneumoniae [23]. However, that study did not find panophthalmitis to be a significant risk factor for ENEV. In contrast, our study found that panophthalmitis was a significant risk factor for ENEV. In various case reports and short series on the management of panophthalmitis, evisceration and enucleation are the most common outcomes [24,25,26,27,28,29,30,31]. In another study of endogenous panophthalmitis, 9 out of 15 patients had had an evisceration; 5 of them had had prior intravitreal treatment that could not control their infection [32]. It has been suggested that the intense inflammatory response to bacterial endotoxins breaks down various ocular layers, thereby facilitating the spread of the infection, that cannot be further controlled [25].

In our study, the initial visual acuity of NLP was significantly associated with ENEV. A prior study from Malihi et al. described 38 cases of corneal ulcer-related infectious endophthalmitis, of which 65.8% of the described patients had NLP visual acuity, 50% were primarily enucleated, and 15.8% became NLP after an average of approximately 4.2 days of treatment [11]. Malihi et al. noticed that, within this group, immunocompromised patients (those having HIV, those having diabetes, and those undergoing chemotherapy) and patients who delayed medical care were more likely to end up being treated with enucleation due to disease progression to a more advanced stage, even when microbiological findings of those who delayed care were similar to those who did not [11]. In another study, Gao et al. found that the proportion of enucleations in a group of patients with corneal ulcer-related infectious endophthalmitis was the highest because of delayed visit time and poor pre-therapy visual acuity, with no subsequent significant improvement to said acuity [5]. Initial visual acuity has been suggested to be used as a predictor of the visual outcome and prognosis of endophthalmitis. Multiple studies have shown that early treatment and good initial visual acuity are important predicting factors for a final prognosis of endophthalmitis [5,9,13]. Similarly, as studies have found a correlation between initial visual acuity and prognosis, there is an association between the causative organisms of infectious endophthalmitis such as Gram-negative bacteria and fungal isolates, which tend to lead to poor visual outcomes [5,33].

Limitations of the study include the presence of an ascertainment bias and a referral bias, as well as the small ample size. Further studies could allow us to obtain a broader picture of the prognosis and outcome(s) of endophthalmitis in Puerto Rico and in the management of such infection.

In conclusion, our study suggests that eyes with infectious endophthalmitis that have a visual acuity of NLP, orbital involvement, and/or exogenous etiology at the initial evaluation have a higher risk of requiring ENEV. Furthermore, eyes with infectious endophthalmitis that have a postsurgical etiology may be at a lower risk of requiring ENEV.

## Figures and Tables

**Table 1:** Demographic and Clinical Characteristics of Study Population.

Number of patients (n)	69
Median age (y)	70 (range, 10–95)
Gender (%)	
Female	40.58%
Male	59.42%
Past Medical History (%)	
Diabetes	62.32%
Immunosuppressed state	4.35%
IVDA history	2.90%
Systemic surgery	2.90%
Eye-Specific Characteristics at Presentation (%)	
Unilateral	100%
Right eye affected	55.07%
Left eye affected	44.93%
Visual Acuity at Presentation (%)	
NLP	24.64%
LP	27.54%
HM	21.74%
CF or more	21.74%
Unable to assess	4.35%
Etiology (%)	
Postsurgical	37.68%
Exogenous	33.33%
Post-traumatic	14.49%
Endogenous	13.04%
Unknown etiology	1.45%
Presence of panophthalmitis (%)	
Panophthalmitis	20.29%
Outcome (%)	
Evisceration or enucleation	46.38%
Eye conservation	53.62%

IVDA: intravenous drug abuse; OD: right eye; OS: left eye; NLP: no light perception; LP: light perception; HM: hand motion; CF: counting fingers.

**Table 2:** Comparison of Characteristics of ENEV Group vs. EP Group.

	ENEV	EP	OR	95% CI	*p* Value
**N**	32 (46.38%)	37 (53.62%)			
**Demographic characteristics**					
**Mean Age**	71.84	65.6		0.82613–13.826	0.071 ^a^
**Male**	16 (50%)	25 (67.57%)	0.485	0.161–1.418	0.216 ^b^
**Female**	16 (50%)	12 (32.43%)			
**Etiology**					
**Postsurgical**	7 (21.88%)	19 (51.35%)	0.271	0.0783–0.848	0.023 ^c^
**Exogenous**	18 (56.25%)	5 (13.51%)	7.946	2.273–33.135	0.0005 ^c^
**Endogenous**	2 (6.25%)	7 (18.92%)	0.291	0.027–1.695	0.161 ^b^
**Post-traumatic**	5 (15.63%)	5 (13.51%)	1.182	0.244–5.738	1 ^b^
**Past Medical History**					
**Diabetes**	19 (59.38%)	24 (64.86%)	0.794	0.267–2.352	0.836 ^b^
**Immunosuppressed state**	1 (3.13%)	2 (5.41%)	0.553	0.009–11.116	1 ^b^
**IVDA History**	1 (3.13%)	1 (2.70%)	1.098	0.014–88.88	1 ^b^
**Clinical characteristics**					
**Time of symptom onset to presentation**					
**Equal or less than 2 days**	6 (18.75%)	16 (43.24%)	0.318	0.085–1.058	0.066 ^b^
**More than 2 days**	24 (75.00%)	20 (54.05%)			
**Visual acuity at presentation**					
**NLP**	15 (46.88%)	2 (5.41%)	17.832	3.476–180.530	0.00003 ^b^
**LP**	7 (21.88%)	12 (32.43%)	0.667	0.187–2.232	0.642 ^c^
**HM**	4 (12.59%)	11 (29.73%)	0.383	0.0785–1.517	0.1497 ^b^
**CF or better**	3 (9.38%)	12 (32.43%)	0.2453	0.0397–1.060	0.044 ^b^
**Final diagnosis of panophthalmitis or endophthalmitis**					
**Panophthalmitis**	12 (37.50%)	2 (5.41%)	10.15	1.967–102.461	0.002 ^b^
**Endophthalmitis**	20 (62.50%)	35 (95.59%)			
**Culture Information**					
**Positive culture**	16 (50.00%)	15 (40.54%)	1.458	0.509–4.239	0.586 ^c^
**Bacteria**	16 (50.00%)	14 (37.84%)	1.631	0.567–4.784	0.4396 ^c^
**Fungus**	0 (0.00%)	1 (2.70%)	0	0.000–45.063	1 ^b^
**Gram+**	10 (31.25%)	12 (32.43%)	0.346	0.0273–2.701	0.3898 ^b^
**Gram−**	5 (15.63%)	2 (5.41%)			

OR: odds ratio; CI: confidence interval; IVDA: intravenous drug abuse, OD: right eye; OS: left eye; NLP: no light perception; LP: light perception; HM: hand motion; CF: counting fingers; EP: eye preserved. ^a^ Welch’s *t*-test. ^b^ Fisher’s exact test (for count data). ^c^ Pearson’s chi-square test.

## Data Availability

The data presented in this study are available on request from the corresponding author.
